# Supplementary material for: Protein secondary structure in spider silk nanofibrils
Source: Nat Commun. 2022 Jul 28;13:4329. doi: 10.1038/s41467-022-31883-3 (PMC9334623; doi:10.1038/s41467-022-31883-3)
Supplement: Supplementary file 1 — Supplementary Information [file 41467_2022_31883_MOESM1_ESM.pdf]

Protein Secondary Structure in Spider Silk Nanofibrils

— *Supplementary Information* —

*Qijue Wang[1], Patrick McArdle[2], Stephanie L. Wang[2], Ryan L. Wilmington[2], Zhen Xing[2], Alexander Greenwood[1], Myriam L. Cotten[1], M. Mumtaz Qazilbash[2], Hannes C. Schniepp[1]**

1. Department of Applied Science, William & Mary, P.O. Box 8795, Williamsburg, VA, 23187-8795, USA.
2. Department of Physics, William & Mary, P.O. Box 8795, Williamsburg, VA, 23187-8795, USA.
**E-mail: schniepp@wm.edu*

1. Experimental setup

Supplementary Figure 1 shows the experimental setup for the polarized vibrational spectroscopy measurements. For p-FTIR, only one polarizer is required before the sample to polarize the incoming IR light. For p-Raman, the laser source is internally polarized and an analyzer was placed after the sample to distinguish scattering signals with different polarizations.

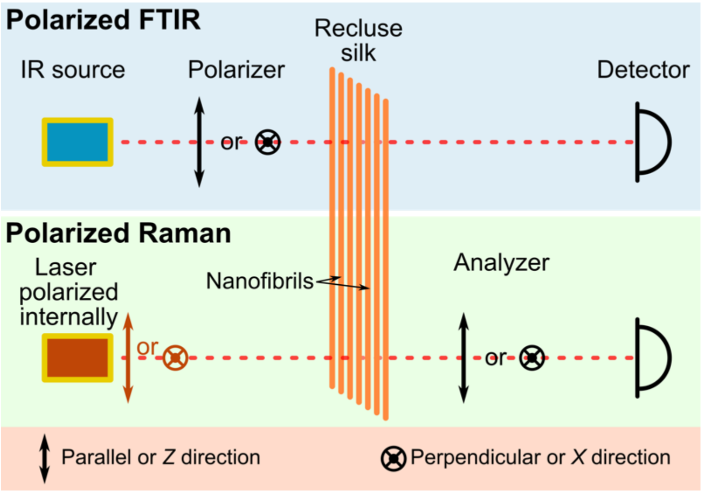

Supplementary Figure 1. Setup schematics of polarized FTIR (light blue background) and Raman (light green background) experiments.

The parallel ("*Z*") direction represents the axial direction of the silk ribbon. Vertical double-headed arrows: red: laser polarization directions for "*Z*"; black: polarizer/analyzer directions for "*Z*". The perpendicular ("*X*") direction represents the direction perpendicular to both the axial and thickness direction of the silk ribbon. Circles with dot and cross in the middle: red: laser polarization directions for "*X*"; black: polarizer/analyzer directions for "*X*".

2. Raman spectra with different polarizations

Supplementary Figure 2 features the complete set of p-Raman spectra, with panels (a) and (b) also featured in Figures 2c and 2d in the main text. Comparing *XX* and *YY* spectra shows that they are almost identical, with several additional weak peaks around 1725 cm^{-1}, 1500 cm^{-1}, and 1150 cm^{-1} observed in the *YY* spectrum.

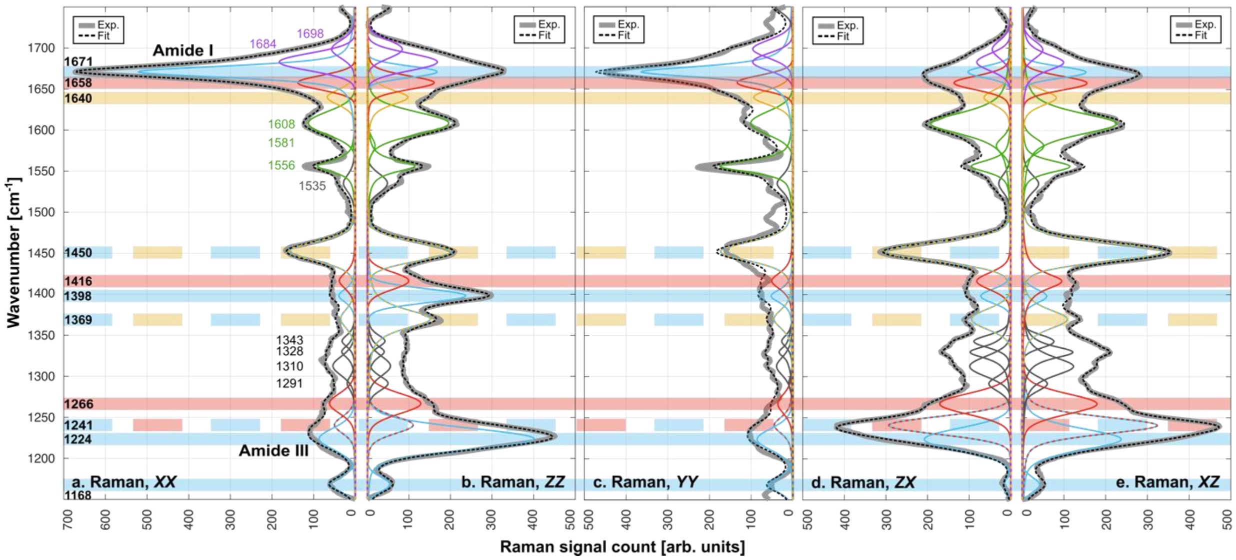

Supplementary Figure 2. p-Raman spectra of all polarization configurations.

(a) *XX* direction; (b) *ZZ* direction; (c) *YY* direction; (d) *ZX* direction; (e) *XZ* direction. The *YY* and *XX* spectra are similar across the measured wavenumber range, where the *YY* spectrum features a lower intensity. *ZX* and *XZ* spectra are nearly identical. Thick gray lines: experimentally (Exp.) measured spectra. Dashed black line: multi-peak fit; all constituting sub-peaks shown in colors. Blue, purple, red, and yellow peaks are assigned to β-sheet, β-turn, helical, and random coil peaks, repsectively. Green peaks represent amino acid residues with aromatic side-chain groups. Gray peaks are unassigned. The colored horizontal bars represent known peaks from the literature, with colors matching the secondary structure assignment.

3. Wide range FTIR and Raman spectra

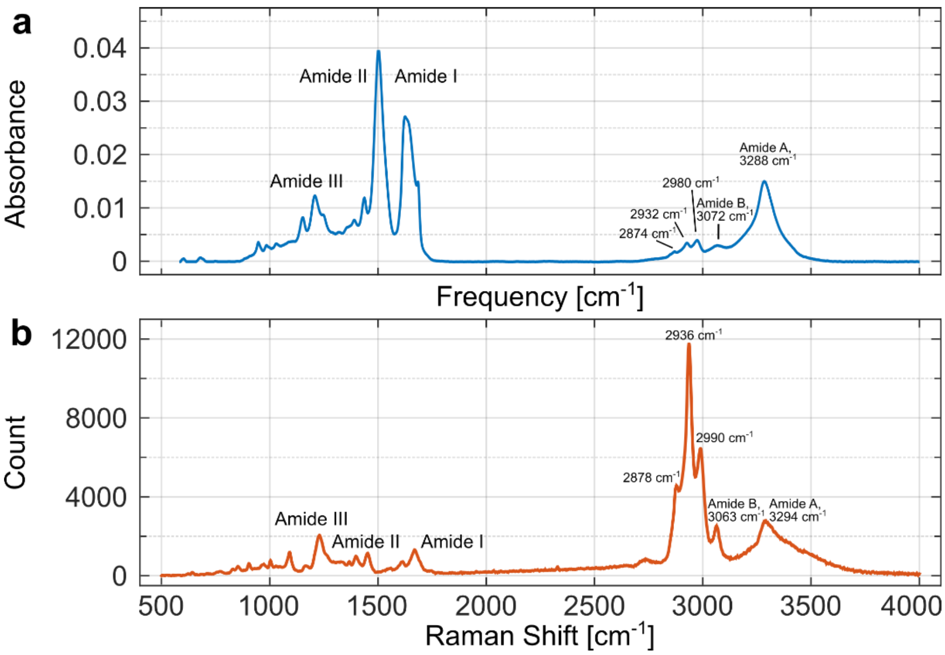

Supplementary Figure 3. Unpolarized FTIR and Raman spectra.

(a) Unpolarized, multi-strand FTIR spectrum and (b) single-strand Raman spectrum of *Loxosceles* silk over 500–4000 cm^{-1}. Amide-I/II/III, A, and B bands and some major peaks in 2800–3100 cm^{-1} are labeled with peak positions.

Supplementary Figure 3 features FTIR and Raman spectra of *Loxosceles* silk ribbon in the 500–4000 cm^{-1} range. Two distinct groups of peaks, one from ≈700–1700 cm^{-1} and one from ≈2800–3300 cm^{-1}, can be identified in these wide-range spectra. It is clear that the relative intensities among amide bands are significantly different between FTIR and Raman results.

4. Peaks related to aromatic groups

In Supplementary Table 1, we summarized the Raman peaks related to aromatic groups and reference peak positions of several aromatic amino acids.

Loxosceles [cm^{-1}]	Phenylalanine [cm^{-1}]	Tyrosine [cm^{-1}]	Tryptophan [cm^{-1}]
1608	1605	≈1600	–
1581	1585	≈1590	1582
1556	–	–	1553

Supplementary Table 1. Summary of observed Raman peaks related to aromatic groups in *Loxosceles* silk. Reference positions for phenylalanine, tyrosine, and tryptophan are provided in columns 2–4 (data from Ref. 1).

5. Error estimation of P_{ZX} values

In order to estimate the standard deviation of the Raman P_{ZX} values featured in Figure 4 in the main text, we introduced uniformly distributed errors to the initial fitting parameters for the sub-peaks. The maximum error caused by the noise generated for the peak position, FWHM, peak magnitude, and Lorentzian content were limited to ± 5 cm^{-1}, ± 5cm^{-1}, 10%, and 0%–100%, respectively. Multiple optimization tests (n = 15) have been made to fit the experimental Raman spectra. The error bars in Figure 4a represent the standard deviation for each Raman P_{ZX} value.

In order to estimate the standard deviation associated with the absorbance subpeaks in the p-FTIR data, we minimized the mean squared error (MSE) between the model and experimental data using the Levenberg-Marquardt algorithm. The process was done using the proprietary package WVASE32. The amplitude and bandwidth of the oscillators in the dielectric function were varied to produce a minimum in the MSE. The error bound on these fits was calculated by evaluating the standard 90% confidence limit (SCL). The uncertainty in the P values can be calculated by propagating the uncertainty:

$$\sigma_P = \sqrt{\left(\frac{\partial P}{\partial |Z|}\right)^2 \sigma_Z^2 + \left(\frac{\partial P}{\partial |X|}\right)^2 \sigma_x^2} \qquad (1)$$

Using the form $P = \frac{|Z|-|X|}{|Z|+|X|}$ above in equation (1), we obtained the uncertainty in the P values and presented them in Figure 4a in the main text as error bars.

6. Orientation distribution of different secondary structures

Based on the symmetry of the silk ribbon and on the knowledge of the natural spinning process of a silk fiber, we assume that any orientation mechanism generates preferred alignment relative to one of the Cartesian axes (X, Y, or Z). Imperfect orientation, thus leads to a distribution of angles around this Cartesian axis, which we assume to be Gaussian ($N(\sigma^2)$). Based on our polarization characterization in a given plane (ZX, ZY, or XY), we can then estimate the standard deviation σ of this Gaussian distribution from the corresponding P value in this plane. For example, in the ZX plane:

$$(2) \qquad |Z| = 2\int_0^\infty A^2 \cos^2\theta N(\sigma^2) d\theta$$

$$(3) \qquad |X| = 2\int_0^\infty A^2 \sin^2\theta N(\sigma^2) d\theta,$$

where A and θ are the dipole moment/polarizability A and its angle θ from the axis. Since $P_{ZX} = \frac{|Z| - |X|}{|Z| + |X|}$, we can numerically find the σ that gives the experimentally observed P_{ZX}. From the expression of $N(\sigma^2)$, we can derive that the half width at half maximum (HWHM) is $\sqrt{2 \cdot \ln(2)}\sigma$. For positive P_{ZX} (preferred alignment with the Z axis), this distribution is around the Z axis; for negative P_{ZX}, this angular distribution is relative to the X axis. According to our calculations, HWHM becomes $>90^o$ for $P<0.0284$ (very weak orientation preference). The HWHM values for the secondary structure related peaks calculated from p-Raman and p-FTIR spectra are featured in Supplementary Tables 2 and 3.

**Structure**	**Peak position, Vibration mode**	P_{ZX}	**HWHM** (around Z for P_{ZX} >0)	P_{ZY}	**HWHM** (around Z for P_{ZY} >0)	P_{XY}	**HWHM** (around X for P_{XY} >0)
β-sheet	Amide-I, 1671 cm^{-1} (CO s)	−0.51	39.4º	−0.38	46.7º (around Y)	0.15	65.6º
	Amide-III, 1224 cm^{-1} (NH ib, CN s)	0.64	31.6º	0.65	31.6º	0.00	> 90º
	1398 cm^{-1} (H^{α} b)	0.69	29.0º	0.65	31.5º	−0.08	75.5º (around Y)
Helix	Amide-I, 1658 cm^{-1} (CO s)	0.02	> 90º	0.11	71.0º	0.09	74.2º
	1416 cm^{-1} (CH_2 b)	0.40	45.7º	0.30	52.3º	−0.11	70.7º (around Y)
	Amide-III, 1266 cm^{-1} (Mixed)	0.36	48.5º	0.57	36.0º	0.26	55.1º
β-sheet + helix	1241 cm^{-1} (Mixed)	0.39	46.5º	0.46	42.2º	0.09	74.9º
Amorphous	Amide-I, 1640 cm^{-1} (CO s)	0.17	63.9º	0.04	86.8º	−0.13	68.0º (around Y)
β-sheet + amorphous	1450 cm^{-1} (CH_3 ab)	0.11	70.7º	0.07	78.5º	−0.04	84.2º (around Y)
	1369 cm^{-1} (CH_3 sb)	0.60	34.0º	0.53	37.8º	−0.10	72.4º (around Y)

Supplementary Table 2. Half width at half maximum (HWHM) values for the secondary structure orientation distribution in the *ZX*, *ZY*, and *XY* planes, calculated from p-Raman spectra. Abbrivations: s, stretching; b, bending; ib: in-plane bending; sb, symmetric bending; ab, antisymmetric bending.

Structure	Band	Peak [cm^{-1}]	Vibration mode	P_{ZX}	HWHM (around Z for P_{ZX} >0)
β-sheet	Amide-I	1699	CO s	0.65	31.2º
	Amide-I	1633	CO s	−0.53	38.0º (around X)
	Amide-II	1517	NH ib, CN s	0.55	36.7º
	—	1406	H^{α} b	0.39	46.2º
	Amide-III	1218	NH ib, CN s	0.57	35.6º
Helix	Amide-III	1265	Mixed	0.42	44.3º
β-sheet + amorphous	—	1450	CH_3 ab	0.17	64.0º
	—	1366	CH_3 sb	0.07	77.4º
	Amide-III	1236	NH ib, CN s	0.30	52.0º
Helix + amorphous	Amide-I	1654	CO s	0.03	> 90º
	Amide-II	1545	NH ib, CN s	−0.12	69.6º (around X)

Supplementary Table 3. Half width at half maximum (HWHM) values for the secondary structure orientation distribution in the *ZX* plane, calculated from p-FTIR spectra. Abbrivations: s, stretching; b, bending; ib: in-plane bending; sb, symmetric bending; ab, antisymmetric bending.

7. Transition dipole coupling (TDC) in antiparallel $(Ala)_n$ β-sheet

In the amide-I band, the FTIR peaks at 1697 and 1631 cm^{-1} demonstrated inverse dichroism (Figures 2a and 2b of the main text). It has been proposed that they are two split peaks caused by transition dipole coupling (TDC) between neighboring C=O groups on adjacent peptide strands in antiparallel β-sheets.[1–3] The associated inverse dichroism can be explained with their corresponding vibration modes (Supplementary Figures 4a–d, adapted from Ref. 4): The 1698 cm^{-1} peak corresponds to the $\nu(0,\pi)$ mode (Supplementary Figure 4b), which has a net component parallel to the β-sheet direction, Z'.[3,4] On the contrary, the 1631 cm^{-1} peak corresponds to the $\nu(\pi,0)$ mode (Supplementary Figure 4c), which has a net perpendicular (X') component.[3,4] Since the intrinsic dichroism of the β-sheets and the observed dichroism in *Loxosceles* silk agree, we can conclude that the β-sheets are generally aligned parallel along the nanofibril direction.

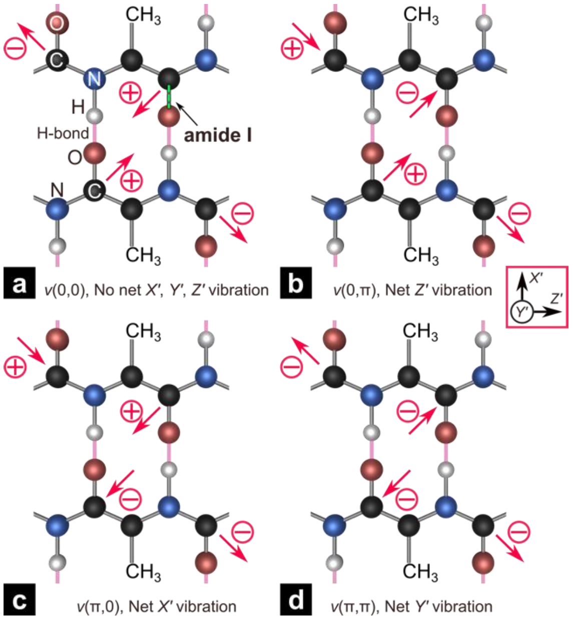

Supplementary Figure 4. Schematics of the transition dipole coupling (TDC) in antiparallel $(Ala)_n$ β-sheets.[4]

Black, blue, red and white spheres represent carbon (C), nitrogen (N), oxygen (O), and hydrogen (H) atoms, respectively. H-bonds are represented by light purple rods and amide-I mode is indicated by the green dashed line. The axes in the red box represent the local orthogonal coordinates. Different modes of C=O stretching vibration are indicated with red arrows (X'– Z' component) and +/– signs (Y' component). (a–d) show four different modes, $\nu(0,0)$, $\nu(0,\pi)$, $\nu(\pi,0)$, $\nu(\pi,\pi)$, and their net vibration directions. The α hydrogen atoms are not shown.

8. Intrinsic P_{ZX} value estimation for 3_1- and α-helices

To estimate the intrinsic P_{ZX} values of 3_1- and α-helices, we calculated the oscillator intensities along *Z'* (helix axial direction) and *X* (direction perpendicular to helix axis) directions for different groups, based on previously published atom coordinates. For the 3_1-helices, we employed the coordinates given by Ramachandran et al.[5] For α-helices, we used atom coordinates of the α-helix segment (KANADAFINSFISAAS) of a MaSpI N-terminal domain model 2N3E.[6] The estimated intrinsic P_{zx} values are presented in Supplementary Table 4. When these two helical structures are considered together with an oscillator population ratio of 1, the final P_{zx} values (third row) match with our experimental results (fourth row) relatively well.

Structure	Amide-I	Amide-II	Amide-III
3_1-helix	−0.733<br>(78% CO stretching,<br>22% CN stretching)	0.431<br>(60% NH in-plane-bending,<br>25% CN stretching,<br>15% $C^{\alpha}C$ stretching)	0.894<br>(CH2 twisting and wagging)
α-helix	0.708<br>(CO stretching)<br>(88% CO stretching,<br>12% CN stretching)	−0.641<br>(52% NH in-plane-bending,<br>37% CN stretching,<br>11% $C^{\alpha}C$ stretching)	−0.528<br>(67% NH in-plane-bending,<br>33 % NCα stretching)
Overall	−0.025	−0.21	0.366
Experiment	0.02 ± 0.05<br>(Raman, 1658 cm^{-1})<br>0.03<br>(IR, 1654 cm^{-1})	−0.08<br>(IR, 1545 cm^{-1})	0.36 ± 0.02<br>(Raman, 1266 cm^{-1})<br>0.44<br>(IR, 1265 cm^{-1})

Supplementary Table 4. Estimation of intrinsic P_{zx} values for 3_1-helices, α-helices, the combination of the two, and experimental P_{zx} values in the amide-I/II/III regions. The overal P_{zx} values agree with the experimental data. The vibration mode components in each amide band are from Ref. 1.

9. X-ray diffraction measurement of *Loxosceles* silk

To prepare the sample, approximately 40 mg of *Loxosceles* silk collected from both male and female spiders was first pressed into a small thin disk (1 cm diameter, 2 mm thickness). Subsequent X-ray diffraction experiment on the sample was performed on a Bruker APEX DUO diffractometer equipped with an APEX II CCD detector and a microfocus copper K_α source (wavelength λ = 1.54 Å).

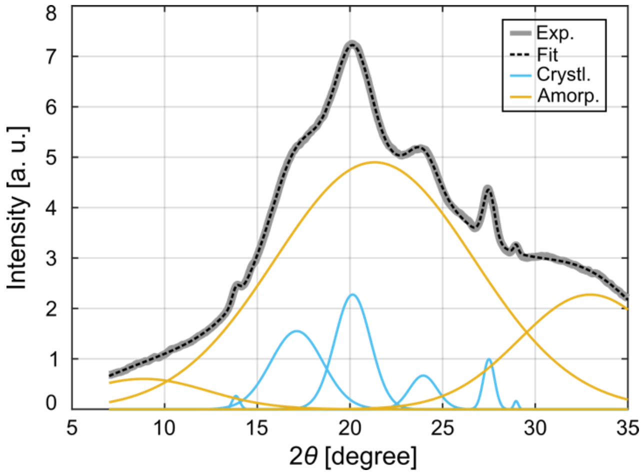

Supplementary Figure 5. X-ray diffraction measurement of *Loxosceles* silk.

A crystallinity is determined to be 43.2%. Gray solid line: experimentally (Exp.) measured spectrum; black dashed line: multi-peak fit; blue solid line: decomposed peaks for crystalline (Crystl.) strcture; yellow solid line: decomposed peaks for amorphous strcture.

To identify the crystal component, we followed the method proposed by Xu et al.:[7] sharp and broad Gaussian peaks are used to fit the experimental data with the least-squares method. The sharp and broad peaks are assigned to crystalline structure and amorphous structure, respectively. The ratio between the sum of crystalline peak magnitudes and the sum of amorphous peak magnitudes is used to represent the crystalline percentage, 43.2%.

10. Calculated unpolarized FTIR spectrum

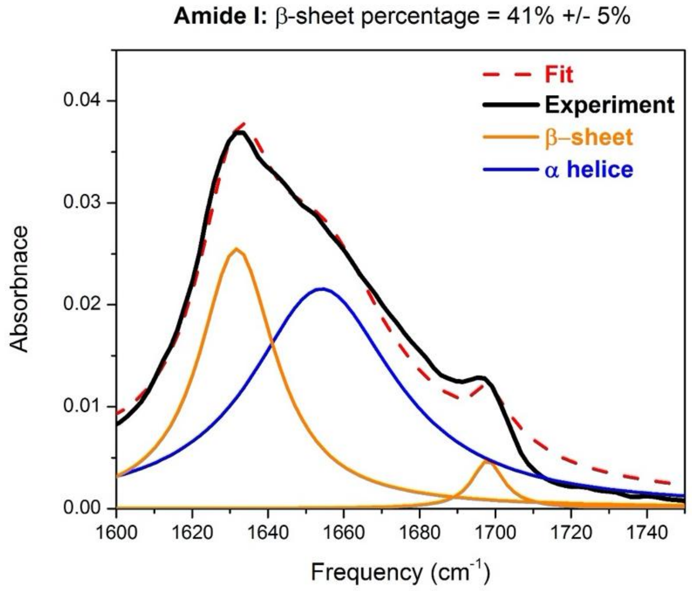

Supplementary Figure 6. Calculated unpolarized FTIR spectrum and its sub-peak decomposition of the amide-I band.

The β-sheet content is determined to be 41±5% from the amide-I band. Black solid line: experimentally measured spectrum; red dashed line: multi-peak fit; yellow solid line: deconvoluted peaks for β-sheet strcture; blue solid line: deconvoluted peak for α-helix strcture.

In order to calculate the β-sheet composition from the FTIR spectra, we used the pseudo-unpolarized spectrum (Supplementary Figure 6). It is necessary to use pseudo-unpolarized data, since the secondary structures are not along only one direction but are instead distributed at different orientations in the plane of the silk. If polarized data was to be used, a different volumetric percentage would be calculated for different polarization directions. We removed the directional dependence of the secondary structures by using the pseudo-unpolarized spectrum, which has no directional dependence. The *Loxosceles* spider silk ribbons are only 50 nm thin in Y direction, orders of magnitude less than in the X and Z directions, and thus, they can be approximated as a quasi-two-dimensional material. Consequently, the Y direction was not considered, and the pseudo-unpolarized spectrum was calculated by averaging the transmission spectra of the parallel (Z) and perpendicular (X) data sets, $(|Z|+|X|)/2$. The β-sheet percentage calculated represents the average β-sheet composition in the ZX plane of the silk. The pseudo-unpolarized absorbance spectrum was deconvolved, using the same methods as described previously for the p-FTIR data sets. The β-sheet composition was calculated for the amide-I

band, by taking the ratio between the area of the β-sheet oscillators to the total area of the amide-I band absorbance. This method of secondary structural composition calculation has often been used in the literature.[8–11]

FTIR spectroscopy on samples with a different geometry may require different methods of calculating pseudo-unpolarized spectra. For instance, for cylindrical fibers, it has been shown that a uniaxial symmetry, and thus $|X|=|Y|$, applies, which leads to $(|Z|+2|X|)/3$ for unpolarized FTIR spectra.[13]

11. Calculated unpolarized Raman spectrum

Calculation of the pseudo-unpolarized spectra is somewhat more complicated for Raman data than for the FTIR spectra discussed in section 10, because there can be up to 16 unknown variables. A mathematically rigorous solution for this problem is challenging and has yet to be demonstrated. We developed the following approximation based on prior work.[12,13] Since we experimentally obtained Raman spectra with the polarizations XX, YY, ZZ, XZ, and ZX, and then found the YY and XX spectra to be similar and with an intensity ratio of 2/3, we assumed $\mathrm{YZ} = \frac{2 \cdot \mathrm{XZ}}{3}$, $\mathrm{ZY} = \frac{2 \cdot \mathrm{ZX}}{3}$, and $\mathrm{XY} = \mathrm{YX} = \frac{\mathrm{XX}+\mathrm{YY}}{2}$ and employed this for an approximation that uses the sum of all nine Raman spectra with different polarization configurations to represent the pseudo-unpolarized Raman spectrum:[12,13]

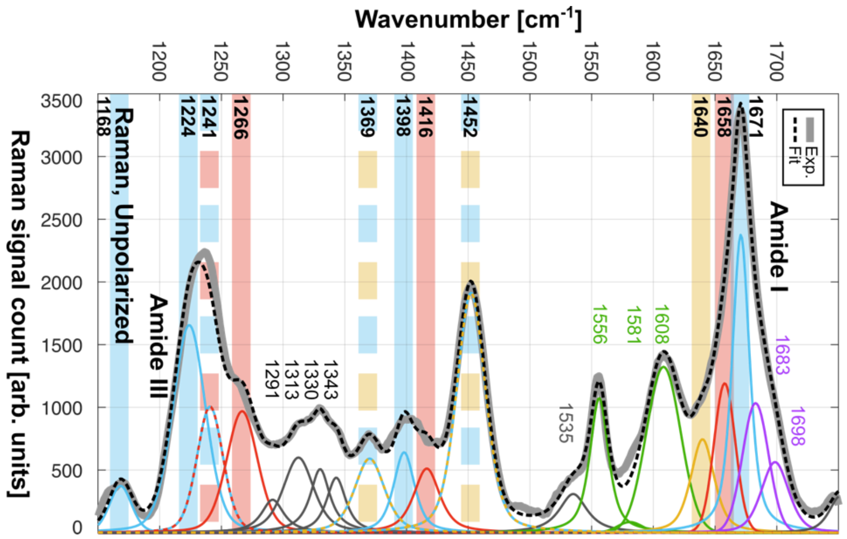

Supplementary Figure 7. Calculated unpolarized Raman spectrum and its sub-peak decomposition. The secondary structure and color assignments are the same as in the Supplementary Figure 2.

$$(4) \qquad \mathrm{Unpolarized} = \mathrm{XX}+\mathrm{YY}+\mathrm{ZZ}+\mathrm{XZ}+\mathrm{ZX}+\mathrm{YZ}+\mathrm{ZY}+\mathrm{XY}+\mathrm{YX}$$

$$= \mathrm{XX}+\mathrm{YY}+\mathrm{ZZ}+\mathrm{XZ}+\mathrm{ZX}+\frac{2(\mathrm{XZ}+\mathrm{ZX})}{3}+(\mathrm{XX}+\mathrm{YY})$$

$$= 2 \cdot \mathrm{XX} + 2 \cdot \mathrm{YY} + \mathrm{ZZ} + \frac{5(\mathrm{XZ}+\mathrm{ZX})}{3}$$

Using the above equation (4), we plotted the calculated unpolarized Raman spectrum (Supplementary Figure 7) and decomposed it with the same set of subpeaks featured in Supplementary Figure 2.

12. Proposed components in mixed peaks

In Supplementary Table 5, we summarize the potential components in the peaks with more than one contributing secondary structure (peaks labeled with dashed multi-color stripes in Figure 2 in the main text).

IR or Raman (yellow) Position [cm^{-1}]	Potential Components
1654	Amorphous, helix
1545	Amorphous, helix
1450	β-sheet, amorphous, aromatic residues
1450	β-sheet, amorphous, aromatic residues
1369	β-sheet, amorphous
1366	β-sheet, amorphous
1241	β-sheet, helix
1236	Amorphous, β-sheet

Supplementary Table 5. Summary of mixed peaks observed in IR and Raman spectra.

13. Ratio of β-sheets and helices based on amide-III analysis

Based on Raman amide-I analysis, the ratio of β-sheets vs helices is 44%/15.9% ≈ 2.8. We looked at the independent Raman amide-III peaks and considered the intensity ratios of three peaks: the β-sheet peak at 1224 cm^{-1}, the helical peak at 1266 cm^{-1}, and the 1241 cm^{-1} peak, which has been proposed to have both helical and β-sheet contributions. The *ZZ* area percentages and P_{ZX} values of these three peaks are {0.65, 0.16, 0.19} and {0.64, 0.36, 0.39}, respectively. The helical component of the last peak is a low-frequency shoulder to the 1266 cm^{-1} peak; its β-sheet component is a high-frequency shoulder to the 1224 cm^{-1} peak. By considering its P_{ZX} values of the three peaks, we estimated that the 1241 cm^{-1} peak is 89% due to helical structures and 11% caused by β-sheets. Using this to calculate the β-sheet vs. helix ratio for all three amide-III peaks, we found 2.0. The same calculation can be carried out for the 3 amide-III FTIR peaks at 1218 cm^{-1} (β-sheet), 1236 cm^{-1} (mixed β-sheet and random coil), and 1265 cm^{-1} (helical), yielding a 55% β-sheet and 45% random coil contributions for the mixed peak. The corresponding β-sheet vs. helix ratio was similar, 1.7.

14. Positions of β-sheet-related peaks in *Loxosceles* silk and several model polypeptides

In Supplementary Table 6, we summarize the β-sheet peak positions in our p-FTIR and p-Raman results, along with the peak positions of β-$(Ala)_n$, β-$(Gly)_n$, and β-$(AlaGly)_n$ in the same region.

IR or Raman (yellow) Position [cm^{-1}]	β-$(Ala)_n$[1]	β-$(Gly)_n$[1]	β-$(AlaGly)_n$[14]
1699	1694	1685	1702
1671	1669	1674	1665
1633	1632	1636	1630
1517	1524	1517	1535
1450	1451	1460	1455
1450	1454, 1446	1432	1450, 1447
1398	1399	1410	1404
1369	1368	–	1369
1366	1372	–	1369
1241	1243	1234	1230
1224	1226	1220	–
1218	1222	1214	–
1168	1165	1162	1161
1168	1167	–	1171

Supplementary Table 6. Summary of observed IR and Raman peaks that are assoicated with β-sheet structure in *Loxosceles* silk, β-$(Ala)_n$, β-$(Gly)_n$, and β-$(AlaGly)_n$. Data for polypeptides adapted from Refs. 1 and 2.

15. Positions of helix-related peaks in *Loxosceles* silk and model polypeptides

In Supplementary Table 7, we summarize the helix peak positions in our p-FTIR and p-Raman results, along with the peak positions of α-$(Ala)_n$ and 3_1-$(Gly)_n$ in the same region.

IR or Raman (yellow) Position [cm^{-1}]	α-$(Ala)_n$[1]	3_1-$(Gly)_n$[1]
1658	1655	1654
1654	1658	1655
1545	1545	1550
1416	–	1421
1406	–	1420
1266	1271	1261
1265	1265	1249
1241	–	1244

Supplementary Table 7. Summary of observed IR and Raman peaks that are assoicated with helical structures in *Loxosceles* silk, α-$(Ala)_n$ (α-helix conformation), and 3_1-$(Gly)_n$. Data for polypeptides adapted from Ref. 1.

16. Summary table

IR Position [cm^{-1}]	Raman Position [cm^{-1}]	Amide group	P_{ZX} Ratio	Structure Assignment	β-sheet mode	Note	Supple-mentary References
	3294	Amide-A	–	NH s			1
3288		Amide-A	–	NH s, possible for β-(Ala)$_n$ and β-(Gly)$_n$			1
3072		Amide-B	–				1,2
	3063	Amide-B	–	NH s			1
	2990	–	–	β-(Ala)$_n$ (CH_3 as)	$\nu(0, 0)$		1
2980			–		$\nu(0, \pi)$		1,14,15
	2936		–	β-(Ala)$_n$ (CH_3 ss)	$\nu(\pi, \pi)$		1
2932			–		$\nu(\pi, \pi)$		1,14,15
	2878		–	β-(Ala)$_n$ ($C_\alpha H_\alpha$ ss)	$\nu(0, 0)$		1
2874			–		$\nu(\pi, 0)$		1,14,15
**Amide-I/II/III regions**							
1699		Amide-I	0.6523 ± 0.1514	β-(Ala)$_n$ (CO s, CN s)	$\nu(0, \pi)$	One of the amide-I split	1,16–20
	1698		0.1258 ± 0.1810	Type I β-turn			12
	1684		−0.0659 ± 0.0603	Type III β-turn			12
	1671		−0.5051 ± 0.0441	β-(Ala)$_n$ (CO s, CNs)	$\nu(0, 0)$	Main Raman peak for β-sheet in amide-I	1,12,17,21
1654			0.0264 ± 0.0289	Amorphous, 3_1- and α-helix			12,22
	1658		0.0203 ± 0.0525	3_1- and α-helix (CO s, CN s, C_αCN d)			
	1640		0.1666 ± 0.0537	Random coil			12
1633			−0.5296 ± 0.0474	β-(Ala)$_n$ (CO s, CNs)	$\nu(\pi, 0)$	One of the amide-I split	1,16,18,20, 23
	1608	– (Aromatic residues)	0.2655 ± 0.0101	Phe, Tyr			1,12,24,25
	1581		0.4883 ± 0.2022	Phe			1
	1556		0.1339 ± 0.0222	Trp			1,26
1545		Amide-II	−0.1188 ± 0.0829	3_1- and α-helix, amorphous			16,26
	1535		0.2376 ± 0.0576	Possible amorphous			16
1517			0.5531 ± 0.0163	β-sheet, possibly formed by glycine rich region (NH ib, CN s, C_αC s, CO ib)	$\nu(0, \pi)$	Observed in β-(Gly)$_n$	1,27
	1450	–	0.1111 ± 0.0115	β-(Ala)$_n$ (CH_3 ab), amorphous, aromatic residues	$\nu(0, 0)$	Higher peak intensity in XZ/ZX	15,17,19,21, 25
1450			0.1658 ± 0.0913		$\nu(0, \pi)$		
	1416		0.3993 ± 0.0403	3_1-helix (CH_2 b)			1,25
1406			0.3922 ± 0.1153	β-(Ala)$_n$	$\nu(0, \pi)$	Raman peak sharper than IR peak	1,14,17,18, 25

	1398		0.6911 ± 0.0266	(H b, CH_3 sb, NH ib)	$\nu(0, 0)$		
	1369		0.6019 ± 0.0217	β-$(Ala)_n$ (CH_3 sb, H_α b), amorphous	$\nu(0, 0)$		1,15,17,18, 25
1366			0.0719 ± 0.2570		$\nu(0, \pi)$		
	1343	Amide-III	0.1848 ± 0.0770	Possible β-$(Ala)_n$ (H_α b, NH ib, C_αC s) or 3_1-helix (CH_2 wg)		Possible peak splitting in Raman	1,9,14,18, 25,28
1338			0.2601 ± 0.3006		$\nu(\pi, \pi)$		
	1328		0.0685 ± 0.0852		$\nu(\pi, \pi)$		
	1310		0.0624 ± 0.0488	Possible β-$(Ala)_n$ (H_α b, CN s, CO ib) or α-helix	$\nu(\pi, \pi)$	Possible peak splitting in Raman	
1301			0.3960 ± 0.3286		$\nu(\pi, 0)$, $\nu(\pi, \pi)$		
	1291		0.3474 ± 0.0778		$\nu(\pi, 0)$		
	1266		0.3557 ± 0.0167	3_1-helix (CH_2 tw), α-helix (NH ib, H_αb)			1
1265			0.4222 ± 0.2175				
	1241		0.3868 ± 0.0279	β-$(Ala)_n$, helices		Strong dichroism in ZX, XZ spectra	1,14,15
1236			0.3041 ± 0.1779	β-$(Ala)_{n,}$ amorphous			
	1224		0.6446 ± 0.0071	β-$(Ala)_n$ (H_α b, NC_α s, NH ib).	$\nu(0,\pi)$	Strong dichroism in ZX, XZ spectra	1,14,15
1218			0.5722 ± 0.2549		$\nu(0,\pi)$		
	1168	–	−0.0343 ± 0.0195	β-$(Ala)_n$ (H_α b, CH3 sb, $C_\alpha C_\beta$ s)	$\nu(0,0)$		1,14,15,18,19
1168			0.6243 ± 0.0754		$\nu(0,\pi)$		
**Additional Peaks**							
1120		–	(*X*)	β-$(Ala)_n$ (CH_3 r, H_αb)	$\nu(\pi, 0)$		1
1106		–	(*Z*)	β-$(Ala)_n$ (NC s, C_α-C_β s)	$\nu(0, \pi)$		1
1097			(*X*)	β-$(Ala)_n$ (CH_3 r, C_α-C_β s)	$\nu(\pi, 0)$		1
	1092	–	–	β-$(Ala)_n$ (CH_3 r, C_α-C_β s)	$\nu(0, 0)$		1,17,21,25
1051			(*X*)	β-$(Ala)_n$ (C_α-C_β s, H_α b)	$\nu(\pi, \pi)$		1,19
1049		–	(*Z*)	$(Pro)_n$-I (C_α-C_β-C_γ s)			19
	1041	–	–	Pro			25
1014			(*X*)	β-$(Gly)_n$ Skeletal stretching			29
	1004	–	–	Aromatic ring breathing, Phe			17,25
1003		–	(*Z*)	$(AlaGly)_n$ CH_3 r			19
	969	–	–	β-$(Ala)_n$ (CH_3 r, NC_α s)	$\nu(0, 0)$		1,17
965		–	–		$\nu(0, \pi)$		1,19

Supplementary Table 8. A summary of observed peaks in pFTIR and pRaman spectra. Abbreviations: s, streching; as, asymmetric streching; ss, symmetric streching; b, bending; ab, asymmetric bending; sb, symmetric bending; ib, in-plane bending; r, rock; d, deformation; tw, twisting; wg, wagging.

Supplementary References

(1) Krimm, S.; Bandekar, J. Vibrational Spectroscopy and Conformation of Peptides, Polypeptides, and Proteins. In *Advances in Protein Chemistry Volume 38*; Elsevier, 1986; pp 181–364. https://doi.org/10.1016/s0065-3233(08)60528-8.

(2) Barth, A. Infrared Spectroscopy of Proteins. *Biochimica et Biophysica Acta (BBA) - Bioenergetics* **2007**, *1767* (9), 1073–1101. https://doi.org/10.1016/j.bbabio.2007.06.004.

(3) Krimm, S.; Abe, Y. Intermolecular Interaction Effects in the Amide I Vibrations of Polypeptides. *Proceedings of the National Academy of Sciences* **1972**, *69* (10), 2788–2792. https://doi.org/10.1073/pnas.69.10.2788.

(4) Miyazawa, T. Perturbation Treatment of the Characteristic Vibrations of Polypeptide Chains in Various Configurations. *The Journal of Chemical Physics* **1960**, *32* (6), 1647–1652. https://doi.org/10.1063/1.1730999.

(5) Ramachandran, G. N.; Sasisekharan, V.; Ramakrishnan, C. Molecular Structure of Polyglycine II. *Biochimica et Biophysica Acta (BBA) - Biophysics including Photosynthesis* **1966**, *112* (1), 168–170. https://doi.org/10.1016/s0926-6585(96)90019-9.

(6) Schaal, D.; Bauer, J.; Schweimer, K.; Scheibel, T.; Roesch, P.; Schwarzinger, S. Amino-Terminal Domain of Latrodectus Hesperus MaSp1 with Neutralized Acidic Cluster. Worldwide Protein Data Bank June 2016. https://doi.org/10.2210/pdb2n3e/pdb.

(7) Xu, D.; Shi, X.; Thompson, F.; Weber, W. S.; Mou, Q.; Yarger, J. L. Protein Secondary Structure of Green Lynx Spider Dragline Silk Investigated by Solid-State NMR and X-Ray Diffraction. *International Journal of Biological Macromolecules* **2015**, *81*, 171–179. https://doi.org/10.1016/j.ijbiomac.2015.07.048.

(8) Fu, F.-N.; Deoliveira, D. B.; Trumble, W. R.; Sarkar, H. K.; Singh, B. R. Secondary Structure Estimation of Proteins Using the Amide III Region of Fourier Transform Infrared Spectroscopy: Application to Analyze Calcium-Binding-Induced Structural Changes in Calsequestrin. *Applied Spectroscopy* **1994**, *48* (11), 1432–1441. https://doi.org/10.1366/0003702944028065.

(9) Cai, S.; Singh, B. R. Identification of *β*-Turn and Random Coil Amide III Infrared Bands for Secondary Structure Estimation of Proteins. *Biophysical Chemistry* **1999**, *80* (1), 7–20. https://doi.org/10.1016/s0301-4622(99)00060-5.

(10) Ling, S.; Qi, Z.; Knight, D. P.; Shao, Z.; Chen, X. Synchrotron FTIR Microspectroscopy of Single Natural Silk Fibers. *Biomacromolecules* **2011**, *12* (9), 3344–3349. https://doi.org/10.1021/bm2006032.

(11) Fang, G.; Tang, Y.; Qi, Z.; Yao, J.; Shao, Z.; Chen, X. Precise Correlation of Macroscopic Mechanical Properties and Microscopic Structures of Animal Silks—Using Antheraea Pernyi Silkworm Silk as an Example. *Journal of Materials Chemistry B* **2017**, *5* (30), 6042–6048. https://doi.org/10.1039/c7tb01638g.

(12) Lefèvre, T.; Rousseau, M.-E.; Pézolet, M. Protein Secondary Structure and Orientation in Silk as Revealed by Raman Spectromicroscopy. *Biophysical Journal* **2007**, *92* (8), 2885–2895. https://doi.org/10.1529/biophysj.106.100339.

(13) Frisk, S.; Ikeda, R. M.; Chase, D. B.; Rabolt, J. F. Rotational Invariants for Polarized Raman Spectroscopy. *Applied Spectroscopy* **2003**, *57* (9), 1053–1057. https://doi.org/10.1366/000370203322258995.

(14) Moore, W. H.; Krimm, S. Vibrational Analysis of Peptides, Polypeptides, and Proteins. II. *β*-Poly(L-Alanine) and *β*-Poly(L-Alanylglycine). *Biopolymers* **1976**, *15* (12), 2465–2483. https://doi.org/10.1002/bip.1976.360151211.

(15) Dwivedi, A. M.; Krimm, S. Vibrational Analysis of Peptides, Polypeptides, and Proteins. XI. *β*-Poly(L-Alanine) and Its N-Deuterated Derivative. *Macromolecules* **1982**, *15* (1), 186–193. https://doi.org/10.1021/ma00229a036.

(16) Paquet-Mercier, F.; Lefèvre, T.; Auger, M.; Pézolet, M. Evidence by Infrared Spectroscopy of the Presence of Two Types of *β*-Sheets in Major Ampullate Spider Silk and Silkworm Silk. *Soft Matter* **2013**, *9* (1), 208–215. https://doi.org/10.1039/c2sm26657a.

(17) Rousseau, M.-E.; Lefèvre, T.; Beaulieu, L.; Asakura, T.; Pézolet, M. Study of Protein Conformation and Orientation in Silkworm and Spider Silk Fibers Using Raman Microspectroscopy. *Biomacromolecules* **2004**, *5* (6), 2247–2257. https://doi.org/10.1021/bm049717v.

(18) Boulet-Audet, M.; Lefèvre, T.; Buffeteau, T.; Pézolet, M. Attenuated Total Reflection Infrared Spectroscopy: An Efficient Technique to Quantitatively Determine the Orientation and Conformation of Proteins in Single Silk Fibers. *Applied Spectroscopy* **2008**, *62* (9), 956–962. https://doi.org/10.1366/000370208785793380.

(19) Papadopoulos, P.; Sölter, J.; Kremer, F. Structure-Property Relationships in Major Ampullate Spider Silk as Deduced from Polarized FTIR Spectroscopy. *The European Physical Journal E* **2007**, *24* (2), 193–199. https://doi.org/10.1140/epje/i2007-10229-9.

(20) Bramanti, E.; Catalano, D.; Forte, C.; Giovanneschi, M.; Masetti, M.; Veracini, C. A. Solid State 13C NMR and FT-IR Spectroscopy of the Cocoon Silk of Two Common Spiders. *Spectrochimica Acta Part A: Molecular and Biomolecular Spectroscopy* **2005**, *62* (1–3), 105–111. https://doi.org/10.1016/j.saa.2004.12.008.

(21) Shao, Z.; Vollrath, F.; Sirichaisit, J.; Young, R. J. Analysis of Spider Silk in Native and Supercontracted States Using Raman Spectroscopy. *Polymer* **1999**, *40* (10), 2493–2500. https://doi.org/10.1016/s0032-3861(98)00475-3.

(22) Kong, J.; Yu, S. Fourier Transform Infrared Spectroscopic Analysis of Protein Secondary Structures. *Acta Biochimica et Biophysica Sinica* **2007**, *39* (8), 549–559. https://doi.org/10.1111/j.1745-7270.2007.00320.x.

(23) Foo, C. W. P.; Bini, E.; Huang, J.; Lee, S. Y.; Kaplan, D. L. Solution Behavior of Synthetic Silk Peptides and Modified Recombinant Silk Proteins. *Appl. Phys. A* **2005**, *82* (2), 193–203. https://doi.org/10.1007/s00339-005-3425-8.

(24) Shao, J.; Zheng, J.; Liu, J.; Carr, C. M. Fourier Transform Raman and Fourier Transform Infrared Spectroscopy Studies of Silk Fibroin. *Journal of Applied Polymer Science* **2005**, *96* (6), 1999–2004. https://doi.org/10.1002/app.21346.

(25) Lefèvre, T.; Paquet-Mercier, F.; Rioux-Dubé, J.-F.; Pézolet, M. Structure of Silk by Raman Spectromicroscopy: From the Spinning Glands to the Fibers. *Biopolymers* **2011**, *97* (6), 322–336. https://doi.org/10.1002/bip.21712.

(26) Monti, P.; Freddi, G.; Bertoluzza, A.; Kasai, N.; Tsukada, M. Raman Spectroscopic Studies of Silk Fibroin from Bombyx Mori. *J. Raman Spectrosc.* **1998**, *29* (4), 297–304. https://doi.org/10.1002/(sici)1097-4555(199804)29:4<297::aid-jrs240>3.0.co;2-g.

(27) Garside, P.; Lahlil, S.; Wyeth, P. Characterization of Historic Silk by Polarized Attenuated Total Reflectance Fourier Transform Infrared Spectroscopy for Informed Conservation. *Applied Spectroscopy* **2005**, *59* (10), 1242–1247. https://doi.org/10.1366/000370205774430855.

(28) Krimm, S. Vibrational Analysis of Conformation in Peptides, Polypeptides, and Proteins. *Biopolymers* **1983**, *22* (1), 217–225. https://doi.org/10.1002/bip.360220130.

(29) Taga, K.; Sowa, M. G.; Wang, J.; Etori, H.; Yoshida, T.; Okabayashi, H.; Mantsch, H. H. FT-IR Spectra of Glycine Oligomers. *Vibrational Spectroscopy* **1997**, *14* (1), 143–146. https://doi.org/10.1016/s0924-2031(96)00061-6.
