# Supplementary material for: Protein secondary structure in spider silk nanofibrils
Source: Nat Commun. 2022 Jul 28;13:4329. doi: 10.1038/s41467-022-31883-3 (PMC9334623; doi:10.1038/s41467-022-31883-3)
Supplement: Supplementary file 3 — Reporting Summary [file 41467_2022_31883_MOESM3_ESM.pdf]

nature portfolio

Corresponding author(s): Hannes Schniepp
Last updated by author(s): Jun 6, 2022

Reporting Summary

Nature Portfolio wishes to improve the reproducibility of the work that we publish. This form provides structure for consistency and transparency in reporting. For further information on Nature Portfolio policies, see our Editorial Policies and the Editorial Policy Checklist.

Statistics

For all statistical analyses, confirm that the following items are present in the figure legend, table legend, main text, or Methods section.

n/a	Confirmed	
☐	☒	The exact sample size (*n*) for each experimental group/condition, given as a discrete number and unit of measurement
☒	☐	A statement on whether measurements were taken from distinct samples or whether the same sample was measured repeatedly
☒	☐	The statistical test(s) used AND whether they are one- or two-sided<br>*Only common tests should be described solely by name; describe more complex techniques in the Methods section.*
☒	☐	A description of all covariates tested
☒	☐	A description of any assumptions or corrections, such as tests of normality and adjustment for multiple comparisons
☐	☒	A full description of the statistical parameters including central tendency (e.g. means) or other basic estimates (e.g. regression coefficient) AND variation (e.g. standard deviation) or associated estimates of uncertainty (e.g. confidence intervals)
☒	☐	For null hypothesis testing, the test statistic (e.g. *F*, *t*, *r*) with confidence intervals, effect sizes, degrees of freedom and *P* value noted<br>*Give P values as exact values whenever suitable.*
☒	☐	For Bayesian analysis, information on the choice of priors and Markov chain Monte Carlo settings
☒	☐	For hierarchical and complex designs, identification of the appropriate level for tests and full reporting of outcomes
☒	☐	Estimates of effect sizes (e.g. Cohen's *d*, Pearson's *r*), indicating how they were calculated

Our web collection on statistics for biologists contains articles on many of the points above.

Software and code

Policy information about availability of computer code

Data collection	Renishaw WiRE 3.4.2377 was used for collection of Raman spectra. Bruker TopSpin 2.1 was used for NMR data collection. Bruker Apex version 3 was used for X-ray data collection. All raw data has been published in the data package at https://doi.org/10.7910/DVN/GMEEYL
Data analysis	MATLAB, version R2018a 64-bit (maci64), and python, version 3.8.5 were used for data analysis (all corresponding codes and scripts are in the data repository published at https://doi.org/10.7910/DVN/GMEEYL). The FTIR data processing software was W-VASE, 3.768. The NMR analysis software was Bruker TopSpin 4.0. GIMP version was 2.10.12. Engauge Digitalizer was version 11.2.

For manuscripts utilizing custom algorithms or software that are central to the research but not yet described in published literature, software must be made available to editors and reviewers. We strongly encourage code deposition in a community repository (e.g. GitHub). See the Nature Portfolio guidelines for submitting code & software for further information.

Data

Policy information about availability of data

All manuscripts must include a data availability statement. This statement should provide the following information, where applicable:

- Accession codes, unique identifiers, or web links for publicly available datasets
- A description of any restrictions on data availability
- For clinical datasets or third party data, please ensure that the statement adheres to our policy

The data that support the findings of this study and the codes used for data analysis are available free of charge at https://doi.org/10.7910/DVN/GMEEYL.

Field-specific reporting

Please select the one below that is the best fit for your research. If you are not sure, read the appropriate sections before making your selection.

☒ Life sciences ☐ Behavioural & social sciences ☐ Ecological, evolutionary & environmental sciences

For a reference copy of the document with all sections, see nature.com/documents/nr-reporting-summary-flat.pdf

Life sciences study design

All studies must disclose on these points even when the disclosure is negative.

Sample size	No sample size calculation was performed. The FTIR and Raman experiments were repeated several times, and the spectra from the repeat experiments were in agreement with the originally measured spectra. Therefore, it was deemed that no further measurements are needed.
Data exclusions	No data was excluded.
Replication	Raman spectra were acquired on samples from 4 different spiders. FTIR spectra were acquired on samples from 2 different spiders. For each sample, spectra were taken on at least 2 different positions. The obtained spectra were similar. NMR and XRD experiments were not replicated because these techniques are much less sensitive. We had to collect the silk from n=30 spiders produced over weeks. However, the NMR and XRD results are thus averaging the data from n=30 different spiders, produced on different days.
Randomization	Randomization does not apply for our experiments, since there were no groups of experiments conducted. All samples contributed equally. Therefore, the order in which the experiments were carried out had no impact on our findings and conclusions. Randomization would not have changed the outcome.
Blinding	Blinding is usually done to prevent observer bias or confirmation bias. However, for our experiments, we found no significant way for the observer to interfere with the measurements. The raw spectra visible to the observer during the experiment are not meaningful without further mathematical analysis. (E.g. determination of peak position via fit routines.) The data analysis routines employed on the spectra post acquisition are strictly deterministic, and we are not aware of any way for the observer to interfere. Therefore, we decided that blinding was not needed for these experiments.

Reporting for specific materials, systems and methods

We require information from authors about some types of materials, experimental systems and methods used in many studies. Here, indicate whether each material, system or method listed is relevant to your study. If you are not sure if a list item applies to your research, read the appropriate section before selecting a response.

Materials & experimental systems

n/a	Involved in the study	
☒	☐	Antibodies
☒	☐	Eukaryotic cell lines
☒	☐	Palaeontology and archaeology
☐	☒	Animals and other organisms
☒	☐	Human research participants
☒	☐	Clinical data
☒	☐	Dual use research of concern

Methods

n/a	Involved in the study	
☒	☐	ChIP-seq
☒	☐	Flow cytometry
☒	☐	MRI-based neuroimaging

Animals and other organisms

Policy information about studies involving animals; ARRIVE guidelines recommended for reporting animal research

Laboratory animals	The silk samples were taken from adult Loxosceles laeta spiders.
Wild animals	The study did not involve any wild animals.
Field-collected samples	The study did not involve sample collected from the field.
Ethics oversight	No ethical was required since spiders are invertebrates.

Note that full information on the approval of the study protocol must also be provided in the manuscript.
